# Anti-Inflammatory Effect of Columbianetin on Lipopolysaccharide-Stimulated Human Peripheral Blood Mononuclear Cells

**DOI:** 10.1155/2018/9191743

**Published:** 2018-04-05

**Authors:** Junying Lu, Keyong Fang, Shiji Wang, Lingxin Xiong, Chao Zhang, Zhongmin Liu, Xuewa Guan, Ruipeng Zheng, Guoqiang Wang, Jingtong Zheng, Fang Wang

**Affiliations:** ^1^Department of Pathogeny Biology, College of Basic Medical Sciences, Jilin University, Changchun 130021, China; ^2^Department of Intensive Care Unit, First Hospital of Jilin University, Changchun 130021, China; ^3^School of Pharmaceutical Sciences, Jilin University, Changchun 130021, China; ^4^Department of Interventional Therapy, First Hospital of Jilin University, Changchun 130021, China

## Abstract

Dysregulated inflammation is increasingly considered as the main cause of many diseases on which NOD1/NF-*κ*B pathway plays an important role. Columbianetin (CBT) is derived from the root of the Chinese herb *Radix Angelicae Pubescentis* for treating inflammatory diseases. Although the anti-inflammatory effect of CBT has been reported, its anti-inflammatory mechanism was poorly studied. In this study, we explored the anti-inflammatory pathway of CBT in lipopolysaccharide- (LPS-) stimulated human peripheral blood mononuclear cell (PBMC) model. Inflammatory cytokine production in culture supernatant was assessed using ELISA assay, and the possible anti-inflammatory pathway of CBT was screened using qPCR array and enrichment analysis with DAVID6.8. To further confirm the targeted pathway of CBT, we pretreated PBMC with the selective NOD1 inhibitor ML130 and then measured the protein levels of the pathway by Western blotting. The result showed that CBT effectively suppressed the expressions of TNF-*α*, IL-6, MCP-1, and IL-1*β* in a dose-dependent manner and significantly downregulated 19 out of 32 differentially expressed genes, most of which were involved in the NOD1/NF-*κ*B pathway, and also showed that CBT remarkably inhibited LPS-induced NOD1, RIP2, and NF-*κ*B activation. Furthermore, the inhibitory effects of CBT on NOD1/NF-*κ*B pathways were blocked by ML130. These findings indicated that CBT inhibits the production of inflammatory cytokines induced by LPS involved in the downregulation of NOD1/NF-*κ*B pathways.

## 1. Introduction

Inflammation is a protective response against infection, tissue stress, and injury in any tissue, and defends and restores physiological functions, but dysregulated inflammatory process could result in chronic inflammation, which is increasingly seen as a major driver of numerous diseases such as obesity, asthma, atherosclerosis, and type 2 diabetes [1, 2]. Nonsteroidal anti-inflammatory drugs (NSAIDs) and corticosteroids are most widely used for the treatment of inflammation; however, the adverse effects of long-term use were unavoidable [3, 4]. Thus, treatment of inflammatory diseases is a big problem. Although a large number of medicinal herbs have been identified as effective anti-inflammatory drugs in the past, but the mechanism of anti-inflammatory herbal compounds has not been elucidated comprehensively [5].

Lipopolysaccharide (LPS), an endotoxin mainly present in the outer membrane of gram-negative bacteria, is the major cause of inflammation. LPS is recognized by specific host pattern-recognition receptors (PRRs) mainly including toll-like receptors (TLRs) and nucleotide-binding and oligomerization domain- (NOD-) like receptors (NLRs) and then induces the activation of NF-*κ*B and mitogen-activated protein kinase (MAPK) signaling pathway, which is involved in the release of various inflammatory cytokines [6, 7]. NOD1, a member of NLRs family, initiates inflammation by recognizing ligands of bacterial peptidoglycan containing *γ*-d-glutamyl-meso-diaminopimelic acid (iE-DAP), which leads to the activation of NF-*κ*B and subsequently the release of proinflammatory mediators and chemokines via a series of cascade responses [8–10]. Dysregulation of NOD1 function has been described in a variety of chronic inflammatory disorders [11]. Therefore, it has been suggested that inhibitors of NOD1 may be useful as anti-inflammatory agents [12].

Columbianetin (CBT) is one of the main bioactive constituents isolated from the root of *Radix Angelicae Pubescentis* (RAP), which has been widely used in China for a long history as an important component in various prescriptions for treating diseases such as arthritis and asthma [13]. As a member of furocoumarin, numerous investigations have shown that CBT has multiple bioactivities, for example, antioxidative [14], antiproliferation [15], anti-inflammatory [16, 17], and anti-nitric oxide production activities [18]. Although the bioeffects of CBT have been reported, the action mechanism has not been well studied.

In the present study, we aimed to explore the anti-inflammatory pathway of CBT. We used the inflammation model of LPS-stimulated human peripheral blood mononuclear cells (PBMCs) and investigated whether CBT could significantly inhibit LPS-induced cytokine production. We then screened CBT targeted pathway by quantitative real-time polymerase chain reaction (qPCR) array and enrichment analysis, which was further confirmed using NOD1 selective inhibitor ML130.

## 2. Methods

### 2.1. Chemicals and Reagents

Lipopolysaccharide (LPS) and 3-(4,5-dimethylthiazol-2-yl)-2,5-diphenyltetrazolium bromide (MTT) were purchased from Sigma-Aldrich (St. Louis, MO, USA); RPMI medium modified without calcium nitrate was purchased from HyClone (Logan, UT, USA); fetal bovine serum (FBS) was obtained from Gibco (Australia); protein assay kit was purchased from BioTime Biotechnology (Shanghai, China); ELISA kits of human TNF-*α*, IL-1*β*, IL-6, and MCP-1 were purchased from RayBiotech (Atlanta, USA); RNeasy Mini Kit, RNase-Free DNase Set, RT^2^ First Strand Kit, RT^2^ SYBR® Green ROX qPCR Master Mix, and Human Inflammasomes PCR Array were obtained from QIAGEN (Valencia, CA, USA). Minute total protein extraction kit was purchased from Invent Biotechnologies (Eden Prairie, USA). NOD1 antibody and RIP2 antibody were purchased from Cell Signaling Technology (Massachusetts, USA). Anti-I*κ*B-alpha antibody and anti-NF-*κ*B antibody were purchased from Abcam (Cambridge, United Kingdom); secondary antibodies were obtained from Sigma-Aldrich (St. Louis, MO, USA). CBT was purchased from Shanghai Yuanye Bio-Technology Company (Shanghai, China; purity ≥ 98%) and ML130 (Abcam, Cambridge, United Kingdom) was prepared in DMSO and diluted with 10% RPMI-1640. Ten percent RPMI-1640 with 0.1% DMSO was used as a vehicle in control group.

### 2.2. Isolation of PBMC

PBMC was isolated from heparinized venous blood via Ficoll density gradient centrifugation method. Heparinized venous blood obtained from the normal donors was diluted 1 : 1 with sterile phosphate-buffered saline (PBS), layered over Ficoll-Hypaque, and centrifuged at 1500 rpm for 15 min at room temperature. PBMC was collected from the interphase layer and washed with PBS twice. The cell viability of isolated PBMC was measured by trypan blue exclusion assay. Then PBMC was resuspended at 1 × 10^6^ cells/ml in RPMI-1640 medium containing 10% charcoal-filtered fatal bovine serum and was kept at 37°C with 5% CO_2_.

### 2.3. Inflammation Model of LPS-Stimulated PBMC and Treatment

Isolated PBMC was divided into 7 groups, including blank control group (Control), LPS-stimulated group (LPS), columbianetin-treated group (LPS + 10, 20, 40 *μ*g/ml CBT), ML130-pretreated group (LPS + ML130), and ML130 pretreatment combined with the treatment of 40 *μ*g/ml columbianetin (LPS + ML130 + CBT). The inflammation model and validation of LPS-stimulated PBMC were established according to a previously described protocol [19]. PBMC was stimulated with 1000 ng/ml LPS for 1 h, then received administrations of different concentrations of CBT (0, 10 *μ*g/ml, 20 *μ*g/ml, 40 *μ*g/ml, resp.) for an additional 24 h, while the other two groups were incubated with 30 *μ*mol/l NOD1 inhibitor (ML130) for 4 h prior to the stimulation of 1000 ng/ml LPS and then followed by the treatment with or without 40 *μ*g/ml CBT. Culture supernatants were collected and stored at −20°C until ELISA assays. Intracellular proteins were collected and stored at −20°C until Western blotting analysis.

### 2.4. ELISA Assay of TNF-*α*, IL-1*β*, IL-6, and MCP-1

The concentrations of TNF-*α*, IL-1*β*, IL-6, and MCP-1 in culture supernatants were quantified using enzyme-linked immunosorbent assay (ELISA) kits (RayBiotech, Atlanta, USA) according to the manufacturer's instructions.

### 2.5. Inflammation-Related Gene Expression Measurement Using qPCR Array

The expression of 84 inflammation-related genes was evaluated using quantitative real-time PCR array (SABiosciences, Valencia, CA) according to the instructions. Total RNA was isolated using the RNeasy Mini Kit and then was quantified and qualitied by measuring the absorbance at 260 and 280 nm. Total RNA was purified with DNase. cDNA synthesis was performed by reverse transcription of 20 ng total RNA as described for RT-PCR and then combined with the SYBR Green Master Mix in 96-well plates following the manufacturer's recommendations. Thermal cycling was performed using an ABI Prism SDS 7300 system (Applied Biosystems, Madrid, Spain).

### 2.6. Enrichment Analysis and the Pathway of Differentially Expressed Genes

Gene expression was analysed using the ∆∆CT method. Screening of differentially expressed genes was based on the standard fold change (FC) ≥ 2 or fold change ≤ 0.5. To investigate the pathway of CBT-targeted inflammatory genes in LPS-stimulated PBMC, enrichment analysis of KEGG pathways was conducted in DAVID6.8 [20]. The significance of the enrichment was determined by *P* value, and meanwhile, the significance of the *P* value was evaluated with false discovery rate (FDR). The pathways were screened in the differentially expressed genes using the enrichment scores (−Log10 (*P* value)). And the significant pathways were screened in accordance with *P* < 0.05 and FDR < 0.05.

### 2.7. Western Blotting Analysis

After treatment, the cells were collected and lysed in RIPA buffer. The same amounts of proteins (25 *μ*g protein per lane) were loaded onto SDS-PAGE gels separated by 12% SDS-PAGE and then were transferred onto PVDF membranes. The membranes were then blocked with 5% fat-free dry milk and probed with the primary antibody at 4°C for 24 h. After washing the membranes thrice, the membranes were incubated with appropriate secondary antibodies for 2 h. Finally, a coloration solution mixture (Beyotime, Jiangsu, China) was added, and the immunoreactive bands were visualized with ECL chemiluminescent detection.

### 2.8. Statistical Analysis

The experimental data were expressed as the means ± standard deviation. Kruskal-Wallis ANOVA or a Mann–Whitney *U* test to compare differences in cytokines and the expression of different proteins among different groups was used in the current study, and a Dunn-Bonferroni test for post hoc comparisons was also used. *P* < 0.05 was considered as statistically significant.

## 3. Results

### 3.1. Effects of CBT on Cytokine Production in LPS-Stimulated PBMC

**Figure 1:**
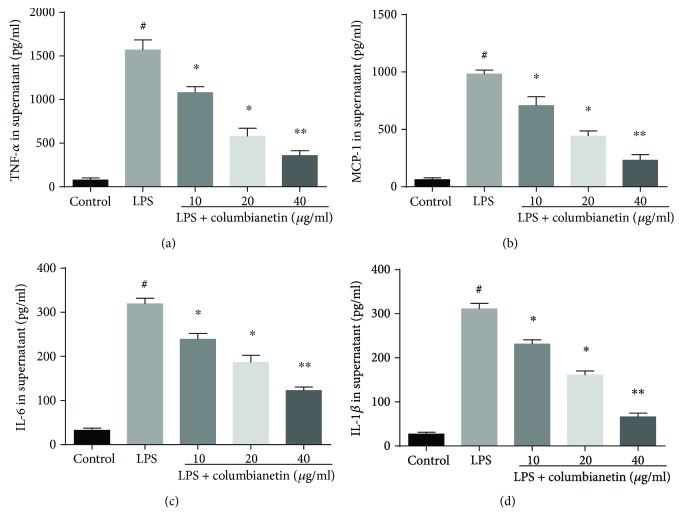
Effects of CBT on TNF-*α*, MCP-1, IL-6, and IL-1*β* production in the supernatant of LPS-stimulated PBMC. The values of three independent experiments are presented as mean ± standard deviation (SD) (*n* = 3 in each group). ^#^ *P* < 0.01 versus control group, ^∗^ *P* < 0.05 versus LPS group, ^∗∗^ *P* < 0.01 versus LPS group.

To assess the anti-inflammatory effects of CBT, cytokine expression of LPS-stimulated PBMC was measured by ELISA. As shown in Figure 1, LPS induced a significant release of TNF-*α*, IL-6, MCP-1, and IL-1*β*. However, treatment with different concentrations of CBT significantly inhibited LPS-induced inflammatory cytokines (TNF-*α*, IL-6, MCP-1, and IL-1*β*) in a dose-dependent manner.

### 3.2. Effects of CBT on the Regulation of Inflammation-Related Genes

**Figure 2:**
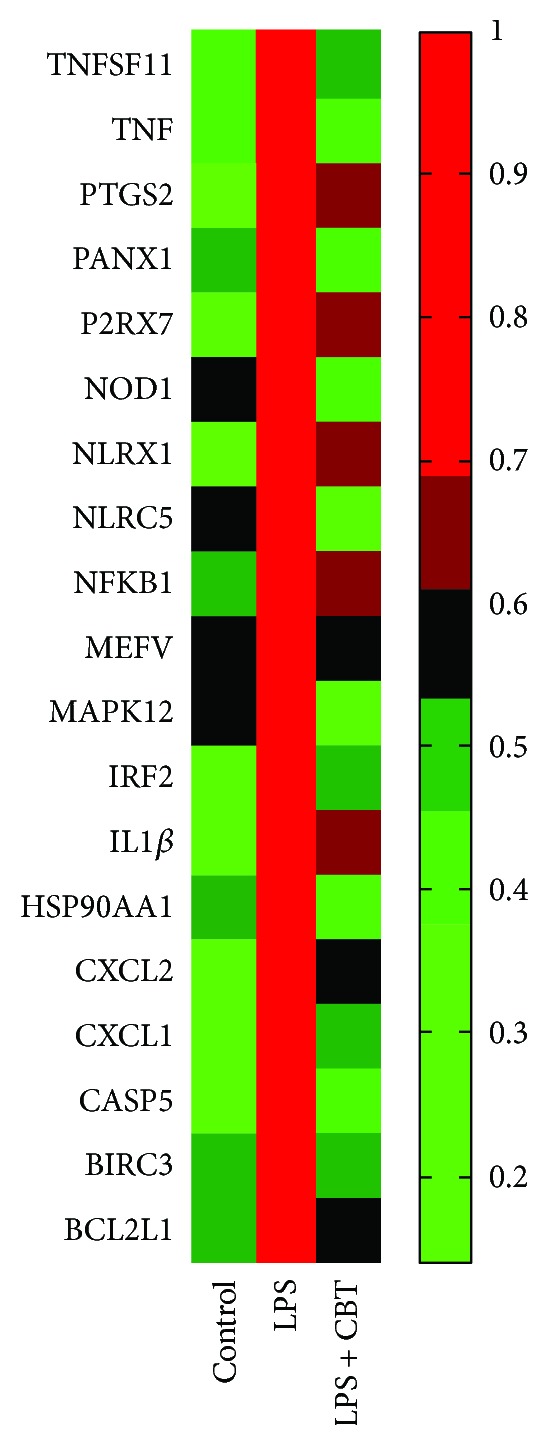
Effects of CBT (40 *μ*g/ml) on the regulation of differential inflammatory-related genes.

In our present study, we found that CBT could inhibit TNF-*α*, MCP-1, IL-6, and IL-1*β* production. To further investigate the possible anti-inflammatory molecular mechanism of CBT, the expression of 84 inflammatory-related genes in PBMC was measured using quantitative real-time polymerase chain reaction (qPCR) array, and then CBT-targeted inflammatory genes were analysed using MATLAB analysis technique. As shown in Supplemental Table 1, LPS caused the differential expression of 44 genes, in which 32 (approximately 72.73%) were upregulated. However, the treatment with CBT significantly downregulated 19 genes which were upregulated by LPS (fold change > 1.5) (Figure 2 and Table 1).

### 3.3. CBT Targeted Pathways of Differentially Expressed Genes

**Figure 3:**
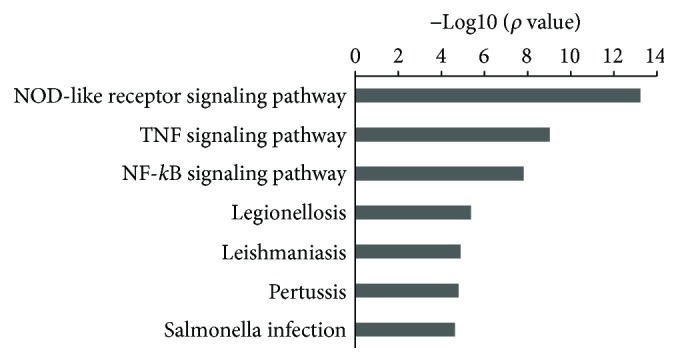
Effects of CBT on the related pathway. The differentially expressed genes of LPS-stimulated human PBMC treated with 40 *μ*g/ml CBT were enriched with DAVID6.8. The black bars showed the pathways in which the differentially expressed gene was found to be involved in the treatment of CBT. The bar plot “−Log10 (*P* value)” represents the enrichment score of the significant enrichment pathways. And the *P* value denotes the significance of the correlation between the pathway and the treatment of CBT.

To elucidate the candidates for CBT targeted pathways, we performed enrichment analysis for the differentially expressed genes. As shown in Figure 3, the differentially expressed genes in LPS-stimulated PBMC pretreated with CBT were enriched in 7 pathways. According to Figure 3, the differentially expressed genes were mainly involved in the NOD-like receptor signaling pathway and demonstrated the highest enrichment score.

### 3.4. Effects of CBT on NOD1/RIP2/NF-*κ*B Pathway

**Figure 4:**
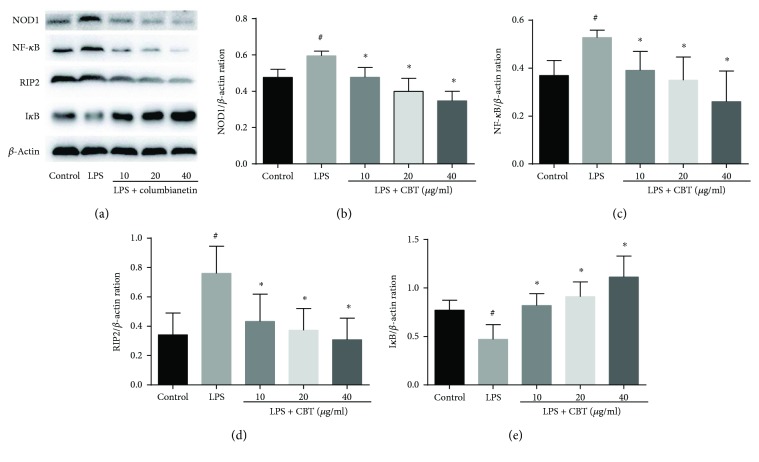
Columbianetin-inhibited LPS-stimulated NOD1/RIP2/NF-*κ*B pathway. The values of three independent experiments are presented as mean ± SD. ^#^ *P* < 0.05 versus control group, ^∗^ *P* < 0.05 versus LPS group.

We found that CBT could downregulate the genes involved in NOD-like receptor signaling pathway. The effects of CBT on NOD-like receptor signaling pathway were measured using Western blot. As shown in Figure 4, after LPS stimulation, the expression of NOD1, RIP2, and NF-*κ*B was dramatically higher compared with the control group, while the expression of I*κ*B*α* was lower. However, after the pretreatment with different concentrations of CBT, the expression of NOD1, RIP2, and NF-*κ*B was evidently reduced and in contrast the expression of I*κ*B*α* was remarkably increased.

### 3.5. Effects of CBT and ML130 on NOD1 of LPS-Stimulated PBMC

**Figure 5:**
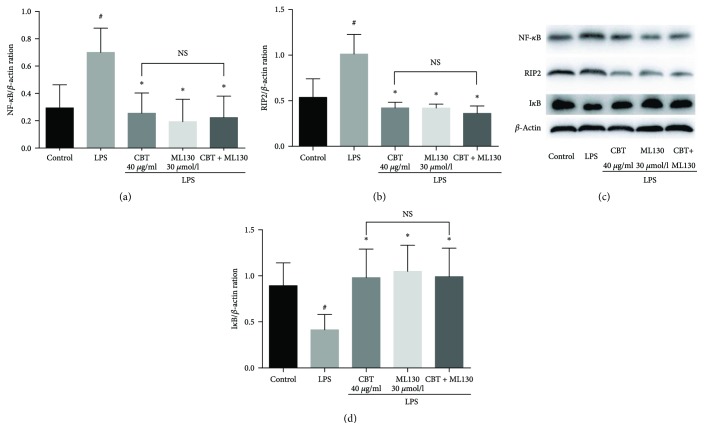
CBT inhibited the expression of NOD1/RIP2/NF-*κ*B pathway by blocking NOD1 activation. The values are presented as mean ± SD of three independent experiments. ^#^ *P* < 0.05 versus control group, ^∗^ *P* < 0.05 versus LPS group. NS indicates no significance among the three groups (*P* > 0.05). CBT: columbianetin.

As shown in Figure 5, the expression of NF-*κ*B and RIP2 was significantly reduced by CBT and ML130 alone to a similar extent (*P* > 0.05). However, the combination of CBT and ML130 did not yield significantly better effects (*P* > 0.05, compared to CBT or ML130 alone, resp.). Our results showed that CBT did not produce any further effect after NOD1 was blocked by ML130. Similarly, ML130 did not further extend the effect of CBT, indicating that these two drugs target the same pathway and exert their anti-inflammatory effects by blocking NOD1 activation.

## 4. Discussion

Inflammatory response is an important pathophysiological process in which NOD1/NF-*κ*B pathway plays a vital role, and dysregulation of inflammation has been identified as a major cause of numerous common diseases [1, 8]. In the present study, we investigated the protective effects and the related pathway of CBT. Our results showed that its anti-inflammation property was exhibited mainly by downregulating NOD1/NF-*κ*B pathway in LPS-stimulated PBMC. Moreover, the selective NOD1 inhibitor ML130 blocked the effect of CBT on NOD1/NF-*κ*B pathway, suggesting the NOD1-dependent mechanism. Our findings indicated that CBT may exert its protective effect mainly through downregulation of NOD1/NF-*κ*B pathway for the first time.

It is well known that LPS can induce inflammatory response that is characterized by elevated proinflammatory cytokines such as TNF-*α*, IL-6, and IL-1*β* [21]. Besides, *in vitro* model of LPS-induced PBMC, TNF-*α*, IL-6, MCP-1, and IL-1*β* was increased notably [19]. A previous study showed that inhibition of these proinflammatory cytokines could attenuate the response of inflammation [22]. Another study also showed that CBT exerted anti-inflammatory effect in LPS-activated RAW 264.7 cells via the inhibition of nitric oxide production [18]. Our results were consistent with previous reports on the anti-inflammatory effects of CBT against LPS-induced inflammation. In the present study, we found that CBT significantly reduced inflammatory cytokine production, which confirmed that CBT had anti-inflammatory effects on LPS-stimulated PBMC.

LPS leads to the release of excessive inflammatory cytokines mainly through the activation of MAPK and NF-*κ*B pathways [6, 7, 23], while the cytokines through the activation of inflammatory and immune cells can regulate the inflammatory response [24]. The MAPK family is comprised by a large group of protein kinases which regulates three major pathways: the extracellular signal-regulated protein kinase 1/2 (ERK), the p38 MAP kinases (p38), and the c-Jun amino-terminal kinase (JNK). And the activation of these reactions is mainly through myeloid differentiation factor 88- (MyD88-) dependent and nondependent TLR4 and NLRS [25]. Moreover, numerous studies show that NLRS and TLR signaling pathways interact at multiple levels in cellular systems [6, 11, 26]. Inflammation induced by LPS is a complex response. As a highly sensitive and reliable method of gene expression profiling, qPCR array combines qRT-PCR and gene chips which can screen differentially expressed genes based on mRNA. qPCR array is a useful tool for studies on signaling pathway [27, 28]. In agreement with these findings, in the current study, the results demonstrated that 44 genes (including MyD88-dependent and nondependent TLR4, NLRS, MARK, and NF-*κ*B) were upregulated after LPS stimulation. To explore the potential mechanism by which CBT exerts its anti-inflammatory effects, the effects of CBT on LPS-stimulated inflammation-associated genes and proteins were assessed. According to the results of qPCR array, CBT downregulated the expression of 19 genes. We repeated the genes which play an important role in the inflammatory response by qPCR, and the results showed that CBT not only downregulated the levels of NOD1/NF-*κ*B, but also downregulated MAPK12 (Supplemental Figure 1); however, the effect on TLR4 was insignificant (Supplemental Figure 2). Moreover, the differentially expressed genes were mainly involved in the NOD1/NF-*κ*B signaling pathway which demonstrated the highest enrichment score. Thus, NOD1/NF-*κ*B was the potential target of CBT in the current evidence; nevertheless, the effects of the drug on MAPK pathway as well as the other molecular pathways need further investigation. We also found that CBT remarkably inhibited the expressions of NOD1, RIP2, and NF-*κ*B. These results indicated that CBT may exhibited its anti-inflammatory effects mainly by inhibiting NOD1/RIP2/NF-*κ*B signaling pathway.

NOD1 has been reported to play an important role in inflammation [8]. Previous studies showed that blocking of NOD1 can alleviate inflammation via suppressing the activation of NF-*κ*B [29] and the release of inflammatory cytokines [9]. Our findings were similar with these results, in which the pretreatment of ML130 significantly suppressed the activation of NF-*κ*B. We have demonstrated that CBT can inhibit the NOD1/NF-*κ*B signaling pathway, and finally, to further confirm that CBT exerts a protective effect mainly through the inhibition of NOD1/NF-*κ*B pathway, we blocked the activation of NOD1/NF-*κ*B pathway using the selective NOD1 inhibitor ML130 and then tested whether CBT continues to influence NOD1/NF-*κ*B signaling pathway. The results showed that CBT did not produce any further effect after NOD1 was blocked. To further confirm whether CBT is a specific NOD1 inhibitor, it is necessary to study the effect of CBT in NOD1 ligand-induced PBMC.

In summary, the results of this study demonstrated that CBT effectively attenuates LPS-induced inflammation at least partially by inhibiting NOD1/NF-*κ*B activities. CBT might be useful as a potential therapeutic medication for LPS-induced inflammation.

## Figures and Tables

**Table 1:** Nineteen significantly downregulated genes in the CBT-treated groups (fold change > 1.5) by CBT.

Number	Gene name	M/N	D/M	Description
1	BCL2L1	2.10	1.74	BCL2-like 1
2	BIRC3	2.03	1.96	Baculoviral IAP repeat containing 3
3	CASP5	2.8	2.54	Caspase 5, apoptosis-related cysteine peptidase
4	CXCL1	3.28	2.02	Chemokine (C-X-C motif) ligand 1
5	CXCL2	2.7	1.88	Chemokine (C-X-C motif) ligand 2
6	HSP90AA1	2.08	2.26	Heat shock protein 90 kDa alpha, class A member 1
7	IL-1*β*	2.82	1.51	Interleukin 1, beta
8	IRF2	1.97	2.07	Interferon regulatory factor 2
9	MAPK12	1.68	3.03	Mitogen-activated protein kinase 12
10	MEFV	1.65	1.85	Mediterranean fever
11	NFKB1	2.1	1.74	Nuclear factor of kappa light polypeptide gene enhancer in B-cell 1
13	NLRC5	1.74	2.96	NLR family, CARD domain containing 5
13	NLRX1	3.51	1.57	NLR family, CARD domain containing 5
14	NOD1	1.76	2.29	Nucleotide-binding oligomerization domain containing 1
15	P2RX7	3.16	1.61	Purinergic receptor P2X, ligand-gated ion channel, 7
16	PANX1	2.20	1.74	Pannexin 1
17	PTGS2	7.1	1.56	Prostaglandin-endoperoxide synthase 2
18	TNF	2.47	2.44	Tumor necrosis factor
19	TNFSF11	2.51	2.01	Tumor necrosis factor (ligand) superfamily, member 11

## Supplementary Materials

Supplementary Materials Supplemental Table 1: 44 differentially expressed genes in LPS-stimulated PBMC (fold change > 1.5). Supplemental Figure 1: columbianetin inhibited the levels of NOD1/RIP2/NF-*κ*B/MAPK12. The values of three independent experiments are presented as mean ± SD. ^#^ *P* < 0.01 versus control group, ^∗^ *P* < 0.05 versus LPS group, ^∗∗^ *P* < 0.01 versus LPS group. Supplemental Figure 2: CBT (40 *μ*g/ml) has no effect on the expression of MYD88 and TIRAP. The values of three independent experiments are presented as mean ± SD. ^#^ *P* < 0.01 versus control group. There is no significance between the groups LPS and LPS + CBT 40 *μ*g/ml.
