# Supplementary material for: Anti-Inflammatory Effect of Columbianetin on Lipopolysaccharide-Stimulated Human Peripheral Blood Mononuclear Cells
Source: Mediators Inflamm. 2018 Apr 5;2018:9191743. doi: 10.1155/2018/9191743 (PMC5907401; doi:10.1155/2018/9191743)
Supplement: Supplementary Materials — Supplemental Table 1: 44 differentially expressed genes in LPS-stimulated PBMC (fold change > 1.5). Supplemental Figure 1: columbianetin inhibited the levels of NOD1/RIP2/NF-κB/MAPK12. The values of three independent experiments are presented as mean ± SD. # P < 0.01 versus control group, ∗ P < 0.05 versus LPS group, ∗∗ P < 0.01 versus LPS group. Supplemental Figure 2: CBT (40 μg/ml) has no effect on the expression of MYD88 and TIRAP. The values of three independent experiments are presented as mean ± SD. # P < 0.01 versus control group. There is no significance between the groups LPS and LPS + CBT 40 μg/ml. [file 9191743.f1.pdf]

Supplemental table 1 44 differentially expressed genes in LPS-stimulated PBMC (Fold Change>1.5)

No.	Gene name	M/N	Description
1	BCL2L1	2.10	BCL2-like 1
2	BIRC3	2.03	Baculoviral IAP repeat containing 3
3	CASP5	2.8	Caspase 5, apoptosis-related cysteine peptidase
4	CXCL1	3.28	Chemokine (C-X-C motif) ligand 1
5	CXCL2	2.7	Chemokine (C-X-C motif) ligand 2
6	HSP90AA1	2.08	Heat shock protein 90kDa alpha, class A member 1
7	IL-1β	2.82	Interleukin 1, beta
8	IRF2	1.97	Interferon regulatory factor 2
9	MAPK12	1.68	Mitogen-activated protein kinase 12
10	MEFV	1.65	Mediterranean fever
11	NFKB1	2.1	Nuclear factor of kappa light polypeptide gene enhancer in B-cell 1
13	NLRC5	1.74	NLR family, CARD domain containing 5
13	NLRX1	3.51	NLR family, CARD domain containing 5
14	NOD1	1.76	Nucleotide-binding oligomerization domain containing 1
15	P2RX7	3.16	Purinergic receptor P2X, ligand-gated ion channel, 7
16	PANX1	2.20	Pannexin 1
17	PTGS2	7.1	Prostaglandin-endoperoxide synthase 2

18	TNF	2.47	Tumor necrosis factor
19	TNFSF11	2.51	Tumor necrosis factor (ligand) superfamily, member 11
20	CD40LG	1.64	CD40 ligand
21	HSP90AB1	1.97	Heat shock protein 90kDa alpha, class B member 1
22	IFNB1	2.1	Interferon, beta 1, fibroblast
23	IFNG	3.7	Interferon, gamma
24	IL12B	5.86	Interleukin 12B
25	IL6	3.58	Interleukin 6 (interferon, beta 2)
26	IRF1	2.44	Interferon regulatory factor 1
27	MAPK1	2.64	Mitogen-activated protein kinase 1
28	NFKBIA	1.98	Nuclear factor of kappa light polypeptide gene enhancer in B-cells inhibitor, alpha
29	NFKBIB	1.59	Nuclear factor of kappa light polypeptide gene enhancer in B-cells inhibitor, beta
30	RIPK2	1.73	Receptor-interacting serine-threonine kinase 2
31	TIRAP	2.13	Toll-interleukin 1 receptor (TIR) domain containing adaptor protein
32	MYD88	2.01	Myeloid differentiation primary response gene (88)
33	CARD6	0.41	Caspase recruitment domain family, member 6
34	CCL2	0.30	Chemokine (C-C motif) ligand 2
35	CCL7	0.07	Chemokine (C-C motif) ligand 7
36	NLRP1	0.60	NLR family, pyrin domain containing 1

37	NLRP9	0.47	NLR family, pyrin domain containing 9
38	NLRP5	0.39	NLR family, pyrin domain containing 5
39	CTSB	0.58	Cathepsin B
40	IL18	0.38	Interleukin 18 (interferon-gamma-inducing factor)
41	NLRC4	0.65	NLR family, CARD domain containing 4
42	NLRP12	0.37	NLR family, pyrin domain containing 12
43	PEA15	0.53	Phosphoprotein enriched in astrocytes 15
44	PYCARD	0.60	PYD and CARD domain containing

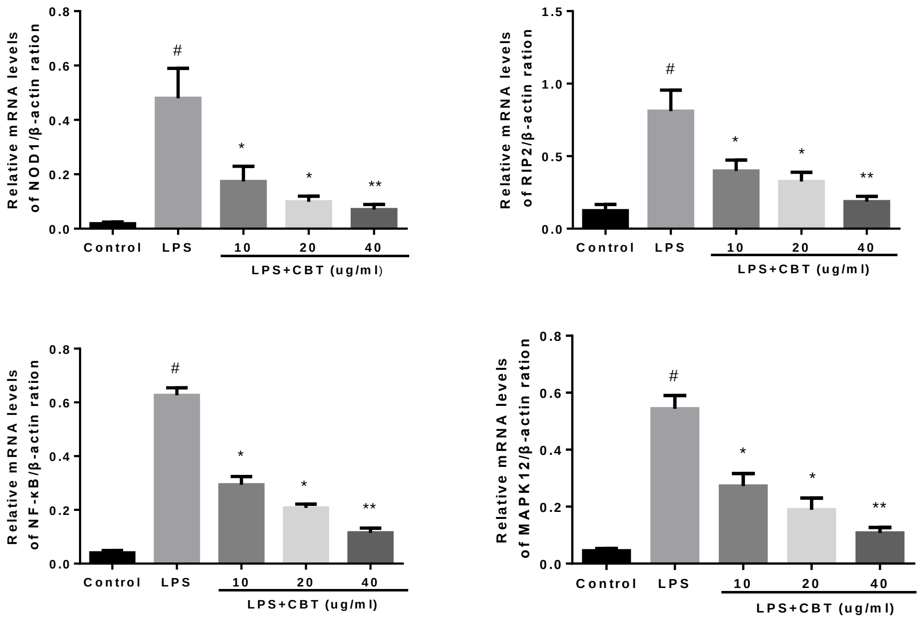

Supplemental Fig. 1 Columbianetin inhibited the levels of NOD1/RIP2/NF-κB/MAPK12. The values of three independent experiments are presented as mean ± SD. # $p < 0.01$ vs. control group, *$p < 0.05$ vs. LPS group, **$p < 0.01$ vs. LPS group.

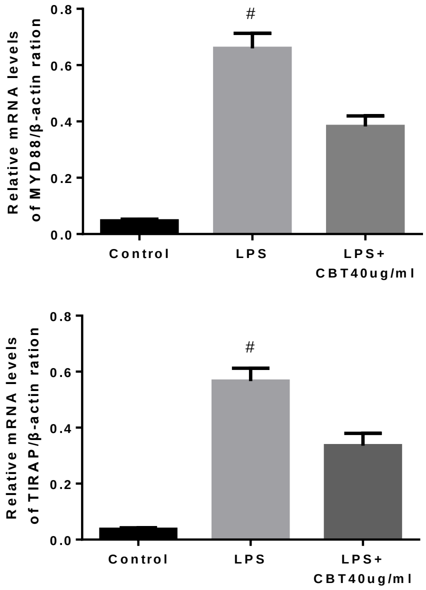

Supplemental Fig. 2 CBT(40ug/ml) has no effect on the expression of MYD88 and TIRAP. The values of three independent experiments are presented as mean ± SD. # $p < 0.01$ vs. control group. There is no significance between the groups LPS and LPS+CBT40ug/ml.
